# Brainstem volume changes in myalgic encephalomyelitis/chronic fatigue syndrome and long COVID patients

**DOI:** 10.3389/fnins.2023.1125208

**Published:** 2023-03-02

**Authors:** Kiran Thapaliya, Sonya Marshall-Gradisnik, Markus Barth, Natalie Eaton-Fitch, Leighton Barnden

**Affiliations:** ^1^National Centre for Neuroimmunology and Emerging Diseases, Menzies Health Institute Queensland, Griffith University, Gold Coast, QLD, Australia; ^2^Centre for Advanced Imaging, The University of Queensland, Brisbane, QLD, Australia; ^3^School of Information Technology and Electrical Engineering, The University of Queensland, Brisbane, QLD, Australia

**Keywords:** myalgic encephalomyelitis/chronic fatigue syndrome, brainstem, magnetic resonance imaging (MRI), pain, breathing difficulty, long COVID

## Abstract

Myalgic encephalomyelitis/chronic fatigue syndrome (ME/CFS) and long COVID patients have overlapping neurological, autonomic, pain, and post-exertional symptoms. We compared volumes of brainstem regions for 10 ME/CFS (CCC or ICC criteria), 8 long COVID (WHO Delphi consensus), and 10 healthy control (HC) subjects on 3D, T1-weighted MRI images acquired using sub-millimeter isotropic resolution using an ultra-high field strength of 7 Tesla. Group comparisons with HC detected significantly larger volumes in ME/CFS for pons (*p* = 0.004) and whole brainstem (*p* = 0.01), and in long COVID for pons (*p* = 0.003), superior cerebellar peduncle (*p* = 0.009), and whole brainstem (*p* = 0.005). No significant differences were found between ME/CFS and long COVID volumes. In ME/CFS, we detected positive correlations between the pons and whole brainstem volumes with “pain” and negative correlations between the midbrain and whole brainstem volumes with “breathing difficulty.” In long COVID patients a strong negative relationship was detected between midbrain volume and “breathing difficulty.” Our study demonstrated an abnormal brainstem volume in both ME/CFS and long COVID consistent with the overlapping symptoms.

## Introduction

Myalgic encephalomyelitis/chronic fatigue syndrome (ME/CFS) is a complex illness that affects multiple body systems and is characterized by a range of symptoms including post-exertional neuroimmune exhaustion (PENE), fatigue, pain, breathing difficulties, and difficulties with concentration and cognitive function (Baker and Shaw, 2007; Carruthers et al., 2011; Stussman et al., 2020). ME/CFS affects 17 to 24 million people worldwide (Lim et al., 2020). There is an absence of a laboratory diagnostic test for ME/CFS, instead diagnosis follows clinical case criteria and exclusion of other illnesses that may account for the symptoms. Over three decades, up to 30 case definitions have been published; however, the three more commonly recognized definitions include Fukuda criteria (Fukuda, 1994), Canadian Consensus Criteria (CCC) (Carruthers et al., 2003), and International Consensus Criteria (ICC) (Carruthers et al., 2011).

Recently, coronavirus 2019 (COVID-19) caused by the novel Severe Acute Respiratory Syndrome Coronavirus 2 (SARS-CoV-2) has infected more than 600 million and caused the deaths of over six million people worldwide (World Health Organization [WHO], 2022). Studies show that up to 43% of people infected by SARS-CoV-2 do not recover fully and develop post-COVID conditions, also known as long COVID (Davis et al., 2021; Chen et al., 2022). Long COVID is defined by the World Health Organisation (WHO) as the continuation or development of new symptoms 3 months after the initial SARS-COV-2 infection, with these symptoms lasting for at least 2 months with no other explanation (World Health Organization [WHO], 2021). The most frequently reported symptoms in the long COVID patients are fatigue, pain, post-exertional malaise, breathing difficulties, and cognitive dysfunction (“brain fog”) (Davis et al., 2021; Komaroff and Bateman, 2021; Mantovani et al., 2021; Nalbandian et al., 2021) that are all common core symptoms of ME/CFS (Davis et al., 2021). Recent studies showed that 13–58% of long COVID patients met ME/CFS criteria (González-Hermosillo et al., 2021; Jason and Islam, 2022; Twomey et al., 2022) and symptoms like fatigue and disability score, autonomic dysfunction, and hand grip strength are similar in ME/CFS and long COVID patients (Kedor et al., 2022). A systematic review of long COVID and ME/CFS has shown that there is a high degree of similarity of fatigue, reduced daily activity, and post-exertional malaise between long COVID and ME/CFS (Wong and Weitzer, 2021). Furthermore, 36.4% of hospitalized COVID-19 patients presented neurological symptoms such as impaired consciousness, dizziness, and headache (Mao et al., 2020). This has stimulated researchers to investigate the effect of SARS-CoV-2 on the central nervous system in long COVID patients.

Magnetic resonance imaging (MRI) is non-invasive, can detect subtle changes in brain structure, and has been used to study brain dysfunction in ME/CFS and COVID patients. Recently, an ME/CFS study demonstrated increased hippocampal subfield volumes (Thapaliya et al., 2022b) and reduced caudal middle frontal volume and precuneus thickness (Thapaliya et al., 2022a). Global differences in gray and white matter volume were observed in ME/CFS (de Lange et al., 2005), although not in all studies (Barnden et al., 2011). Voxel-based morphometry (VBM) reported a decrease in the pons and midbrain volume and an increase in the amygdala and insula volumes in ME/CFS patients (Finkelmeyer et al., 2018). An MRI study in COVID-19 patients showed reduced gray matter thickness in the para-hippocampal gyrus, anterior cingulate cortex, and temporal lobe (Douaud et al., 2022). COVID-19 patients also have higher gray matter volume in the left Rolandic operculum, bilateral olfactory cortices, bilateral insulas, bilateral hippocampi, and right cingulate gyrus (Lu et al., 2020) and lower mean diffusivity in the left insula, cingulate gyri, right precuneus, right thalamus, and superior frontal-occipital fasciculus (Lu et al., 2020). MRI scans before and after COVID-19 infection showed an increased volume in the putamen, temporal cortex, fusiform and para-hippocampal gyrus (Salomon et al., 2021).

Recent studies have shown that COVID-19 survivors will develop symptoms of long COVID in all cohorts, even in young adults, students, children (Greenhalgh et al., 2020; Yelin et al., 2021; Yong, 2021a). Progression from COVID infection into long COVID may result from tissue damage, viral persistence, and/or chronic inflammation that remains unresolved after acute COVID-19 (Baig, 2020; Greenhalgh et al., 2020; Yelin et al., 2021; Yong, 2021a). Another potential cause could be persistent brainstem dysfunction (Yong, 2021b). Autopsy studies in the brainstem of deceased COVID-19 patients have shown shrunken neurons and inflammation (Al-Dalahmah et al., 2020), hemorrhages (Bradley et al., 2020), positive SARS-CoV-2 RNA (Deigendesch et al., 2020; Fabbri et al., 2021), and perivascular and interstitial encephalitis and neurodegeneration (von Weyhern et al., 2020). Notably long COVID symptoms overlap with ME/CFS in which brainstem dysfunction has been reported. The symptom severity of ME/CFS was associated with brainstem dysfunction (Barnden et al., 2016). MRI studies showed lower mean diffusivity (Thapaliya et al., 2021), higher signal intensity (Barnden et al., 2018; Thapaliya et al., 2020), and impaired brainstem connectivity (Barnden et al., 2019) in the brainstem regions of ME/CFS patients. The brainstem regulates respiratory, cardiovascular, gastrointestinal, and neurological processes and its impairment can explain the overlapping symptoms of ME/CFS and long COVID. Brainstem invasion by viruses (Deigendesch et al., 2020; Fabbri et al., 2021), pathological immune, or vascular activation (Al-Dalahmah et al., 2020; Fabbri et al., 2021) might lead to brainstem dysfunction in ME/CFS and long COVID.

Despite several studies showing a similar symptom presentation between ME/CFS and long COVID, structural change in the brainstem using MRI is yet to be investigated. The specific aims of this pilot study were to (a) quantify volumes of brainstem subregions and the whole brainstem in ME/CFS and long COVID and compare them to healthy controls (HC), and (b) explore the relationship between brainstem volumes and clinical symptom severity in ME/CFS and long COVID patients.

## Materials and methods

### Participant recruitment

The study was approved by the Griffith University Human Research Ethics Committee (ID: 2022/666) and written informed consent was obtained from all individuals. This cross-sectional investigation was conducted at the National Centre for Neuroimmunology and Emerging Diseases (NCNED) on the Gold Coast, Queensland, Australia. Eligible participants were contacted using the NCNED research registry database. ME/CFS patients were considered eligible if they fulfilled the CCC and/or ICC definitions for diagnosis, had received a formal diagnosis of ME/CFS by a physician, and did not report a history of COVID-19 infection. Participants with long COVID reported symptoms persisting for at least 3 months following COVID-19 infection according to the WHO working case definition. HC reported no diagnosis of a chronic health condition or evidence of underlying illness and had no current or prior COVID-19 infection. Participants were aged between 18- and 65-years. Medical history was requested to identify comorbid manifestations or exclusionary diagnoses including mental illness, malignancies, autoimmune, neurological, or cardiovascular diseases. Female participants were excluded if they were pregnant and/or breastfeeding. Finally, 10 ME/CFS patients fulfilling the CCC and ICC criteria (Carruthers et al., 2011), eight long COVID as defined by the WHO clinical case definition (World Health Organization [WHO], 2021) and 10 age-matched HC subjects were included in this study (see Table 1 for demographic information).

**TABLE 1:** Demographic and clinical characteristics of patients with ME/CFS, long COVID, and HC.

	ME/CFS (*n* = 10)	Long COVID (*n* = 8)	HC (*n* = 10)	*P*-value
Age	46.4 ± 15.2	43.2 ± 10.7	42.3 ± 14	0.53^a^, 0.29^b^, 0.69^c^,
F/M	6/4	5/3	7/3	N/A
Pain	38 ± 20.4	37.8 ± 16.4	87 ± 19.7	<0.001^a^, <0.001^b^, 0.98^c^
Breathing difficulty	0.8 ± 1.13	1.8 ± 1.6	N/A	0.15^c^

Superscripts a, b, and c are the *p*-values for ME/CFS vs. HC, long COVID vs. HC, and ME/CFS vs. long COVID, respectively.

### Symptom presentation and clinical measures

Symptom presentation was collected using the NCNED Research Registry questionnaire developed by NCNED with the Centres for Disease Control and Prevention (CDC) Symptom Inventory Questionnaire distributed online through LimeSurvey. The presence and severity of each symptom was assessed on a five-point scale: (1) very mild; (2) mild; (3) moderate; (4) severe; and (5) very severe. Validated patient-reported outcome measures were used to determine participant quality of life (QoL) and functional capacity. The 36-item short form health survey (SF-36) (Alonso et al., 1995) has been frequently employed in previous observational studies to assess QoL among people with ME/CFS (Eaton-Fitch et al., 2020), as well as, more recently, among people with the long COVID condition (O’Kelly et al., 2022). Eight QoL domains were assessed including physical functioning, role limitations due to physical health problems, bodily pain, general health perceptions, vitality, social functioning, role limitations due to personal or emotional health, and emotional wellbeing/mental health. Survey item scores were assigned a value between 0 and 100, before scores were averaged for each domain.

For subsequent correlation analysis, the severity measure of “pain” was extracted from SF36v2, while breathing scores were obtained *via* the NCNED Research Registry questionnaire. Symptom severity of 10 ME/CFS and eight long COVID patients have been provided as a Supplementary material.

### MRI scans and data processing

Magnetic resonance imaging was performed on a 7 T whole-body MRI research scanner (Siemens Healthcare, Erlangen, Germany) with a 32-channel head coil (Nova Medical Wilmington, Wilmington, NC, USA). We acquired T1-weighted data using a Magnetization prepared 2 rapid acquisition gradient echo sequence (MP2RAGE) as in Thapaliya et al. (2019). In brief, MP2RAGE data were acquired sagittally using the following parameters: repetition time (TR) = 4,300 ms, echo time (TE) = 2.45 ms, first inversion time (TI1) = 840 ms, TI2 = 2,370 ms, first flip angle (FA1) = 5°, FA2 = 6° and resolution = 0.75 mm^3^ with matrix size = 256 × 300 × 320.

MP2RAGE data were processed similarly to our previous publications (Thapaliya et al., 2022a,b). In brief, MP2RAGE images were anatomically segmented using FreeSurfer version 7.1.1 (Fischl, 2012) ^1^using the default FreeSurfer command “recon-all” on a Macintosh computer (Operating system: Catalina, RAM = 36GB, and core: 8). The “recon-all” processing includes motion correction, non-linear spatial normalization to Talairach space, intensity normalization, removal of non-brain tissue, cortical percolation, sub-cortical segmentation, gray and white matter boundary tessellation, automated topology correction, and surface deformation. Detailed information about the pipeline can be found at.^2^

**FIGURE 1:**
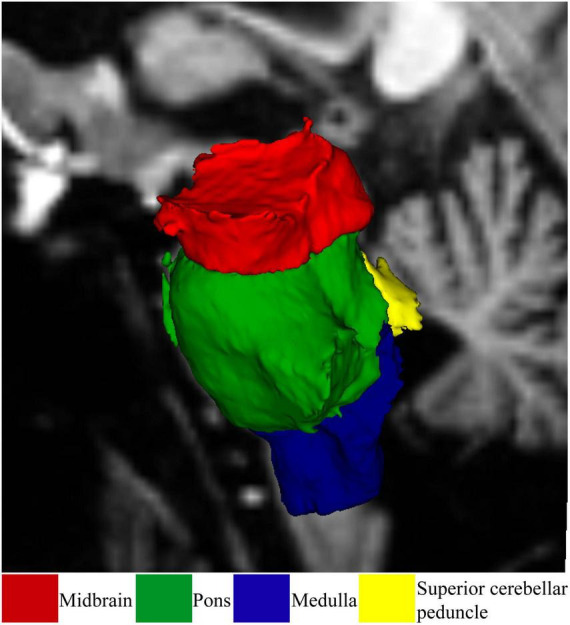
Demonstrates brainstem subregions of a healthy participant. Subregions are color coded.

Brainstem subregions were segmented using the FreeSurfer 7.1.1 brainstem module (Iglesias et al., 2015) as shown in Figure 1. Using this module, the brainstem was segmented into the midbrain, pons, superior cerebellar peduncle (SCP), and medulla oblongata. Brainstem subregions for all participants were visually checked for distortion-free segmentation.

### Statistical analysis

Multivariate general linear model (GLM) statistical analysis was performed to test brainstem subregions and whole brainstem volume differences between ME/CFS, long COVID patients, and HC using SPSS version 28. After confirmation of homogeneity using Levene’s test, the multivariate GLM was used to test for three group differences. Correction for multiple group comparisons was implemented using the Bonferroni method. Then Spearman correlations were performed between brainstem subregion and whole brainstem volumes and clinical severity measures for ME/CFS and long COVID patients. The normality condition for data was checked using the Shapiro-Wilk method available in SPSS before the correlation. Age and sex were included as covariates for group comparisons and correlation analysis.

## Results

### Group comparison: ME/CFS vs. HC

**FIGURE 2:**
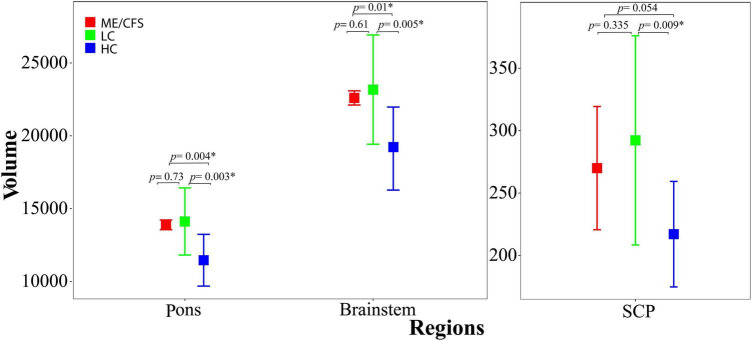
Shows the estimated mean volumes and their standard deviations (bars) for the pons and whole brainstem regions **(left)** and SCP **(right)** across ME/CFS (red), long COVID (green), and HC (blue) participants. ME/CFS and long COVID mean volumes were both significantly larger than HC (*p* < 0.05) in the pons and whole brainstem region. SCP volumes were only significantly larger than HC in long COVID. Error bars indicate one standard deviation. SCP, superior cerebellar peduncle.

The brainstem subregion volumes were *larger* in ME/CFS patients compared with HC (see Table 2). After adjusting for multiple comparisons, volumes remained significantly *larger* in the pons (*p* = 0.004) and whole brainstem (*p* = 0.01) (see Figure 2 and Table 2).

**TABLE 2:** For ME/CFS, and HC, the mean and standard deviation of volumes for the brainstem subfields.

	Volume in mm^3^		*P*-value	95% confidence interval	
				Lower	Upper
Regions					
	ME/CFS	HC			
Medulla	3110.3 ± 155.7↑	2756.1 ± 440.6	0.166	-238.7	830.8
Pons	13889.5 ± 333.5↑	11461.3 ± 1776.9	**0**.004*	444.1	4227.1
Midbrain	5331.6 ± 295.5↑	4792.1 ± 507.1	0.056	-151.5	1220.1
SCP	270.01 ± 83.81↑	217.10 ± 42.25	0.054	-13.2	110.0
Whole brainstem	22601.4 ± 488.7↑	19226.7 ± 2644.3	**0**.01*	261.7	6167.0

↑ Indicates a larger volume in ME/CFS than in HC. *Represents difference from HC statistically significant (*p* < 0.05) after adjusting for multiple comparisons. SCP, superior cerebral peduncle.

### Group comparison: Long COVID vs. HC

In long COVID patients, after adjusting for multiple comparisons, we observed significantly *larger* volumes in the pons (*p* = 0.003), SCP (*p* = 0.009), and whole brainstem (*p* = 0.005) (see Figure 2 and Table 3). The medulla (*p* = 0.042) and midbrain (*p* = 0.026) volumes were not significantly larger compared with HC (see Table 3) after adjusting for multiple comparisons.

**TABLE 3:** Volume means and standard deviations for long COVID and HC for the brainstem subregions and the whole brainstem.

	Volume in mm^3^		*P*-value	95% confidence interval	
				Lower	Upper
Regions					
	Long COVID	HC			
Medulla	3302.4 ± 696.7↑	2756.1 ± 440.6	0.042	-95.0	1052.9
Pons	14120.3 ± 2305.8↑	11461.3 ± 1776.9	**0**.003*	569.0	4629.6
Midbrain	5456.1 ± 889.2↑	4792.1 ± 507.1	0.026	-55.4	1416.7
SCP	292.27 ± 83.81↑	217.10 ± 42.25	**0**.009*	6.8	139.1
Whole brainstem	23171.1 ± 3750.7↑	19226.7 ± 2644.3	**0**.005*	662.5	7001.1

Long COVID volumes were statistically different from HC (*p* < 0.05). ↑ Indicates a larger volume in long COVID than HC. SCP, superior cerebral peduncle, *represents statistical significance after adjusting for multiple comparisons with the Bonferroni method.

### Group comparison: ME/CFS vs. long COVID

Although brainstem subregion volumes were smaller in ME/CFS patients compared with long COVID (see Table 4), these differences were not statistically significant (*p* < 0.05).

**TABLE 4:** Volume means and standard deviations volumes for ME/CFS and long COVID for the brainstem subfields and whole brainstem and their statistical inference.

	Volume in mm^3^		*P*-value	95% confidence interval	
				Lower	Upper
Regions					
	ME/CFS	Long COVID			
Medulla	3110.3 ± 155.7↓	3302.4 ± 696.7	0.407	-741.6	375.9
Pons	13889.5 ± 333.5↓	14120.3 ± 2305.8	0.734	-2240.2	1712.7
Midbrain	5331.6 ± 295.5↓	5456.1 ± 889.2	0.603	-862.9	570.2
SCP	270.01 ± 83.81↓	292.27 ± 83.81	0.335	-88.9	39.8
Whole brainstem	22601.4 ± 488.7↓	23171.1 ± 3750.7	0.610	-3702.7	2467.8

↓ Indicates a smaller volume in ME/CFS patients than long COVID. No significant volumetric differences were obtained between ME/CFS and long COVID. SCP, superior cerebral peduncle.

### Brainstem subregion volume correlations with pain and breathing

**FIGURE 3:**
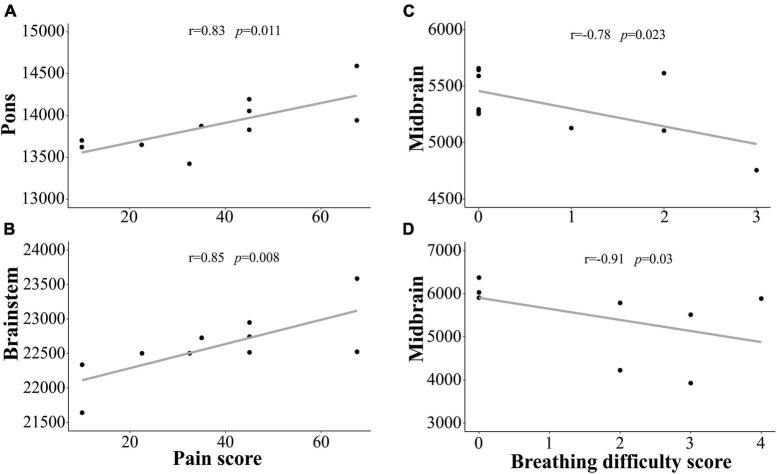
Shows the strong correlation between brainstem region volumes and clinical measures for ME/CFS and long COVID patients. We observed statistically significant relationship between the pons, brainstem volumes and “pain” score in ME/CFS patients **(A,B)**. We also found statistically significant relationship between midbrain volume and “Breathing difficulty” score in ME/CFS **(C)** and long COVID **(D)** patients. *Y*-axis is the volume and *x*-axis are the clinical scores.

We demonstrated that subregion and whole brainstem volumes in ME/CFS and long COVID patients are significantly associated with clinical measures of “pain,” and “breathing difficulty” (see Figure 3 and Table 5). We observed a significantly strong positive relationship between “pain” and volume of pons (*r* = 0.83, *p* = 0.011) and whole brainstem (*r* = 0.85, *p* = 0.008) (see Table 5). There was also a strong negative relationship between “breathing difficulty” and midbrain (*r* = −0.78, *p* = 0.023) and whole brainstem (*r* = −0.78, *p* = 0.022) volumes in ME/CFS patients (see Figure 3). Furthermore, we found a very strong negative relationship between “breathing difficulty” and midbrain volume (*r* = −0.91, *p* = 0.03) in long COVID patients (see Figure 3 and Table 5).

**TABLE 5:** Correlation between brainstem region volumes and clinical measures in ME/CFS and long COVID.

Brainstem region	Clinical measure	*r*	*P*
**ME/CFS**			
Pons	Pain	0.83	0.011
Brainstem	Pain	0.85	0.008
Midbrain	Breathing difficulty	−0.78	0.023
Brainstem	Breathing difficulty	−0.78	0.022
**Long COVID**			
Midbrain	Breathing difficulty	−0.91	0.03

r, correlation coefficient. The Spearman correlation test was used to perform correlation analysis using SPSS software version 28.

## Discussion

This study reports volumetric differences in the whole brainstem and four subregions in ME/CFS, long COVID, and HC. We showed that pons, SCP, and whole brainstem volumes were significantly larger in long COVID patients compared with HC. Similarly, pons and whole brainstem volumes were significantly larger in ME/CFS patients compared with HC. Interestingly, no brainstem subregion volumes were significantly different between ME/CFS and long COVID patients between ME/CFS and long COVID patients. To the authors’ knowledge this is the first investigation to demonstrate the overlap between ME/CFS and long COVID metrics using MRI. We also demonstrated that “pain” and “breathing difficulty” are strongly associated with brainstem volumes in ME/CFS and long COVID.

### Group comparisons

Our study found significantly *larger* volumes for whole brainstem, pons, and SCP in ME/CFS and long COVID patients. The brainstem contains multiple small and dispersed neuron structures in the midbrain, pons, and medulla (Naidich et al., 2009) which together they constitute the reticular activation system (RAS). RAS nuclei connect with each other and to the body and subcortical and cortical structures (Guyton and Hall, 2011). RAS neurons influence cortical function *via* two different pathways. Firstly, RAS neuron projections deliver neurotransmitters directly or indirectly (e.g., *via* hypothalamus, basal forebrain) to the cortex (Saper and Fuller, 2017), and secondly RAS neurons generate oscillatory electrical signals that facilitate the coherence of cortical oscillations necessary for attention, sensory perception, problem solving, and memory (Garcia-Rill et al., 2013). Excitatory midbrain nuclei and inhibitory medulla nuclei constitute a circuit that controls both cortical arousal levels (cognition, wake/sleep, pain, respiration) and gait selection (e.g., walking or running) in response to inputs from multiple brain centers (Stornetta, 2008; Nicholls and Paton, 2009). Therefore, structural changes in the brainstem of ME/CFS and long COVID patients could result in severe and varied deficits in brain function.

### ME/CFS vs. HC group comparison

We observed a larger volume for the whole brainstem and pons in ME/CFS patients compared with HC. Previous studies in ME/CFS patients have reported lower mean diffusivity in the pons (Thapaliya et al., 2021), and higher T1/T2 signal intensity in the medial lemniscus and cortical spinal tract (Thapaliya et al., 2020) that is sensitive to the level of myelination or iron. A functional MRI study reported impaired connectivity within the brainstem and to the hippocampus and thalamus of ME/CFS patients (Barnden et al., 2019). In ME/CFS patients, decreased myelin-sensitive T1-weighted spin echo signals were detected in the brainstem (Barnden et al., 2018) and the brainstem perfusion ratios were reduced (Costa et al., 1995). The brainstem contains the nuclei of the reticular activation system which control arousal, the sleep/wake cycle, gait, and memory *via* cortical connections and cardio-respiratory function (Costa et al., 1995; Garcia-Rill et al., 2013, 2016). Therefore, brainstem dysfunction is consistent with the symptoms experienced by ME/CFS patients including cognitive dysfunction, sleep disturbance, orthostatic intolerance, and dyspnea.

### Long COVID vs. HC group comparison

We also found larger volumes of the whole brainstem, pons, and SCP in long COVID patients compared with HC. Such volume increases may reflect edema of inflammatory responses, neurodegeneration, and/or viral invasion (Yong, 2021b). Autopsy studies of the brain have detected SARS-CoV-2 RNA and proteins in the brainstem of COVID-19 patients (Deigendesch et al., 2020; Matschke et al., 2020). Higher concentrations of SARS-CoV-2 are consistent with the high expression of Angiotensin-converting enzyme 2 which is the receptor SARS-CoV-2 uses to infect host cells in the brainstem (Letko et al., 2020; Zhou et al., 2020). Other autopsy studies showed inflammation, neuronal cell loss, and axonal degeneration in the brainstem of COVID-19 patients (Matschke et al., 2020; von Weyhern et al., 2020). Activated microglia and astrocytes, leukocyte infiltration, and micro-thrombosis have also been reported in the brainstem of COVID-19 patients (Deigendesch et al., 2020; Schurink et al., 2020; Meinhardt et al., 2021; Mukerji and Solomon, 2021). A microscopy study showed more tissue damage in the pons in COVID-19 patients than in controls (Bulfamante et al., 2021), and MRI also showed severe damage to the brainstem in two COVID-19 patients (Manganelli et al., 2020). Abnormal diffusion (lower fractional anisotropy) was reported in the SCP for multiple sclerosis patients with cerebellar symptoms and this correlated with cognitive performance (Nicoletti et al., 2017). The SCP has large sensory and motor nerve tracts that connect the cortex and pons and facilitate refined motor movements, learning of new motor skills, and balance (Khonsary, 2022). However, the function of this region needs to be investigated in different diseases. Damage to the brainstem, in particular the respiratory neurons of the dorsal medulla, could cause respiratory failure which is a key symptom of COVID-19 patients (Boutou et al., 2021a,b; Huang et al., 2021). Brainstem dysfunction has been demonstrated in chronic migraine headache (Aurora and Brin, 2017; Chong et al., 2017) which also occurs in long COVID (Membrilla et al., 2021). Therefore, structural changes in the brainstem are associated with the heterogeneous changes in brain function that correspond to the key symptoms of long COVID.

### ME/CFS vs. long COVID group comparison

We did not find significant differences in the brainstem volumes of ME/CFS and long COVID patients which is consistent with the overlapping presentation of both cohorts (Sukocheva et al., 2021; Marshall-Gradisnik and Eaton-Fitch, 2022). Cardiovascular and respiratory symptoms of ME/CFS and long COVID are controlled by neuronal circuits between the hypothalamus and the brainstem (Benarroch, 2018). The symptom overlap between ME/CFS and long COVID patients is consistent with by our current findings of similar abnormalities in the brainstem. Further, a recent investigation demonstrated the biological overlap of ME/CFS and long COVID through transient receptor potential melastatin 3 (TRPM3) ion channel dysfunction (Sasso et al., 2022). TRPM3 ion channel dysfunction in the pathology of both ME/CFS and long COVID suggests further research is required to determine whether the illnesses are separate. TRPM3 channels are widely expressed through multiple cell and tissue types and are highly expressed in the brainstem, thus may account for a common pathology in ME/CFS and long COVID (Held and Tóth, 2021; Ragozzino et al., 2021).

### Correlations with clinical measures

We detected significant correlations between clinical measures (pain and breathing difficulty) and volumes of the whole brainstem and its subregions in ME/CFS and long COVID patients. Pain is regarded as one of the major symptoms of ME/CFS (Bourke et al., 2014). Our study shows a significantly strong positive correlation between “pain” and pons and whole brainstem volumes in ME/CFS patients (see Figure 3 and Table 5) indicating that larger brainstem volumes are associated with higher pain severity. The brainstem regions have several nuclei that receive ascending and descending signal pathways that inhibit or facilitate pain by upward or downward regulation of neurotransmission (Mills et al., 2021). Several brainstem nuclei including periaqueductal gray in the midbrain, dorsal and median raphe nuclei, parabrachial nucleus, and locus coeruleus in the pons region are involved in pain processing (Napadow et al., 2019). Functional connectivity differences were observed between brainstem nuclei in fibromyalgia patients (Ioachim et al., 2022). Recently, a study showed that the hippocampal subfield volumes were associated with pain levels in ME/CFS patients (Thapaliya et al., 2022b).

Breathing difficulty is another common symptom experienced by ME/CFS and long COVID patients (Ravindran et al., 2013), (Mancini et al., 2021). It has been reported that 30–50% of COVID-19 patients experience breathing difficulty (Mandal et al., 2021; Shah et al., 2021). We showed that smaller midbrain and whole brainstem volumes were associated with more severe “breathing difficulty” in both ME/CFS and long COVID patients (see Figure 3 and Table 5). Breathing difficulties in ME/CFS and long COVID are associated with brainstem volume changes that may reflect changes to the respiratory and cardiovascular neuronal circuits in the brainstem (Benarroch, 2018). The brainstem has a ventral respiratory column that controls rhythmic breathing (Smith et al., 1991; Moreira et al., 2011), a pontine respiratory group that controls the transition between expiration and inspiration (Stornetta, 2008), and the caudal ventrolateral medulla that controls inspiration (Nicholls and Paton, 2009). Therefore, brainstem dysfunction may contribute to the respiratory-related symptoms in ME/CFS and long COVID.

### Limitations

This study does have some limitations. This is a pilot study with a relatively small sample size that will affect the power of the study to detect brainstem volume differences and their association with clinical measures. Another limitation is that pain and breathing scores were obtained using self-reported questionnaires, which by their subjective nature may limit the interpretation of our findings. This study was a cross-sectional study; therefore, further investigations with a larger cohort and longitudinal studies are recommended to test progressive changes in the brainstem volume in ME/CFS and long COVID patients.

## Conclusion

In this pilot study, volumetric differences in brainstem regions were detected in ME/CFS and long COVID patients relative to HC. Clinical measures for “pain” and “breathing difficulty” showed a strong relationship with pons, midbrain, and whole brainstem volumes in ME/CFS and long COVID patients. Interestingly, volumes of the whole brainstem and its subregions were not significantly different between ME/CFS and long COVID patients. This is consistent with ME/CFS and long COVID having similar brainstem abnormalities which will contribute to their neurological and cardio-respiratory symptoms.

## Data availability statement

The original contributions presented in this study are included in this article/Supplementary material, further inquiries can be directed to the corresponding author.

## Ethics statement

The studies involving human participants were reviewed and approved by Griffith University. The patients/participants provided their written informed consent to participate in this study.

## Author contributions

KT: conceptualization, formal analysis, and writing – original draft. KT and LB: methodology. KT, LB, NE-F, MB, and SM-G: writing – review and editing. LB and SM-G: supervision. All authors contributed to the article and approved the submitted version.

## Footnotes

1
https://surfer.nmr.mgh.harvard.edu/

2
https://surfer.nmr.mgh.harvard.edu/fswiki/recon-all
