# Supplementary material for: Brainstem volume changes in myalgic encephalomyelitis/chronic fatigue syndrome and long COVID patients
Source: Front Neurosci. 2023 Mar 2;17:1125208. doi: 10.3389/fnins.2023.1125208 (PMC10017877; doi:10.3389/fnins.2023.1125208)
Supplement: Supplementary file 1 [file Table_1.docx]

Table 1 shows the symptom severity of 8 long COVID patients

| ID | Age | Sex | Duration  Years | Pain | Fatigue | Cognition | Breathing difficulty | Unrefreshing Sleep | Physical  Functioning |
| --- | --- | --- | --- | --- | --- | --- | --- | --- | --- |
| 1 | 49 | F | 0.75 | 22.5 | 4 | 35 | 3 | 3 | 65 |
| 2 | 64 | M | 0.25 | 32.5 | 3 | 45 | 2 | 4 | 75 |
| 3 | 46 | F | 0.17 | 57.5 | 3 | 20 | 0 | 3 | 80 |
| 4 | 30 | F | 0.58 | 47.5 | 3 | 25 | 3 | 3 | 85 |
| 5 | 40 | M | 0.16 | 45 | 3 | 50 | 0 | 4 | 90 |
| 6 | 59 | F | 0.16 | 55 | 3 | 55 | 2 | 3 | 15 |
| 7 | 61 | M | 0.33 | 32.5 | 4 | 80 | 0 | 3 | 75 |
| 8 | 43 | F | 0.25 | 10 | 4 | 50 | 4 | 4 | 10 |

Table 2 shows the symptom severity of 10 ME/CFS patients

| ID | Age | Sex | Duration  Years | Pain | Fatigue | Cognition | Breathing difficulty | Unrefreshing Sleep | Physical  Functioning |
| --- | --- | --- | --- | --- | --- | --- | --- | --- | --- |
| 1 | 26 | F | 2 | 35 | 4 | 50 | 0 | 4 | 65 |
| 2 | 61 | M | 18 | 22.5 | 4 | 35 | 1 | 5 | 75 |
| 3 | 47 | F | 2.5 | 45 | 3 | 40 | 0 | 3 | 35 |
| 4 | 60 | F | 45 | 67.5 | 5 | 65 | 0 | 4 | 10 |
| 5 | 31 | F | 5 | 32.5 | 3 | 70 | 2 | 4 | 80 |
| 6 | 60 | F | 15 | 10 | 5 | 50 | 3 | 4 | 45 |
| 7 | 32 | M | 10 | 45 | 3 | 55 | 2 | 4 | 40 |
| 8 | 64 | M | 3 | 10 | 4 | 45 | 0 | 2 | 45 |
| 9 | 31 | F | 36 | 45 | 4 | 16.7 | 0 | 5 | 20 |
| 10 | 31 | F | 17 | 67.5 | 3 | 10 | 0 | 3 | 65 |

F=Female, M=Male

Fatigue, Breathing difficulty, Unrefreshing sleep severity: 0 =none; 1= very mild, 2 = Mild, 3 =Moderate, 4=severe, 5= very severe

Pain, Cognition, Physical functioning severity: 0 =none; 20= very mild, 40 = Mild, 60 =Moderate, 80=severe, 100= very severe
